# Dermal Telocytes: A Different Viewpoint of Skin Repairing and Regeneration

**DOI:** 10.3390/cells11233903

**Published:** 2022-12-02

**Authors:** Catalin G. Manole, Mihaela Gherghiceanu, Laura Cristina Ceafalan, Mihail E. Hinescu

**Affiliations:** 1 Ultrastructural Pathology Laboratory, Victor Babes National Institute of Pathology, 050096 Bucharest, Romania; 2 Department of Cellular and Molecular Biology and Histology, Carol Davila University of Medicine and Pharmacy, 050474 Bucharest, Romania; 3 Cell Biology, Neurosciences and Experimental Myology Laboratory, Victor Babes National Institute of Pathology, 050096 Bucharest, Romania

**Keywords:** telocytes, telopodes, skin rejuvenation, skin repairing/regeneration

## Abstract

Fifteen years after their discovery, telocytes (TCs) are yet perceived as a new stromal cell type. Their presence was initially documented peri-digestively, and gradually throughout the interstitia of many (non-)cavitary mammalian, human, and avian organs, including skin. Each time, TCs proved to be involved in diverse spatial relations with elements of interstitial (ultra)structure (blood vessels, nerves, immune cells, etc.). To date, transmission electron microscopy (TEM) remained the single main microscopic technique able to correctly and certainly attest TCs by their well-acknowledged (ultra)structure. In skin, dermal TCs reiterate almost all (ultra)structural features ascribed to TCs in other locations, with apparent direct implications in skin physiology and/or pathology. TCs’ uneven distribution within skin, mainly located in stem cell niches, suggests involvement in either skin homeostasis or dermatological pathologies. On the other hand, different skin diseases involve different patterns of disruption of TCs’ structure and ultrastructure. TCs’ cellular cooperation with other interstitial elements, their immunological profile, and their changes during remission of diseases suggest their role(s) in tissue regeneration/repair processes. Thus, expanding the knowledge on dermal TCs could offer new insights into the natural skin capacity of self-repairing. Moreover, it would become attractive to consider that augmenting dermal TCs’ presence/density could become an attractive therapeutic alternative for treating various skin defects.

## 1. Introduction

Considering the skin as the largest human organ, which is completely covering the human body, and taking into account at least a few of its most prominent roles (mechanical protection, physical barrier, etc.), it is natural to consider that skin as being prone to various injuries. These injuries intercept the barrier function and could be either acute or chronic, sometimes emerging abruptly, but also within the context of some dermatological pathologies. Another function of the skin (frequently much augmented) is its social/aesthetic function, since healthy and presentable skin enhances the self-esteem of many individuals, contributing to a better social life [1]. Currently, millions of people are suffering either acute or chronic wounds that need to be therapeutically addressed. Acute wounds include iatrogenic wounds (e.g., surgical injuries) or accidental skin injuries of various severities. Besides these, chronic skin conditions could be physiological (e.g., face skin wrinkles of different extents or atrophic skin) or chronic skin wounds (e.g., associated with diabetes, venous insufficiency, obesity, etc.) [2]. In this context, the estimated wound care products market is increasingly projected every year, with different sources indicating a market value of billions of Euros, and is further elevated in times of extensive accidents, wars, etc. Therefore, finding better options for treating these wounds would improve the patient care systems. Developing new therapeutic protocols for skin wound repair (cell-based) could drastically reduce the financial and economic burden of skin wound care issues, while also enhancing the social impact. Further skin repairing protocols should be in accordance with the new technological advancements and/or developments in cellular and molecular medicine (especially concerning tissue engineering and regenerative medicine). This should become the future policy in skin wound healing management and antiaging dermatology.

However, a better understanding of the cellular and molecular mechanisms underlying skin wound recovery and repair/regeneration after trauma is necessary. Wound care science could investigate this series of interrelated biological events, which are serially developing as the wound is progressing, in several directions [3], all of which would involve a better understanding of the wound microenvironment and the progression chronology of the cellular and molecular response sequences after injury [4]. Establishing novel protocols in accordance with the new technological advancements and/or developments in cellular and molecular medicine (especially regarding tissue engineering and regenerative medicine) should become the future policy in skin wound healing management and antiaging dermatology.

## 2. Telocytes

At least for the histology of human tissues, the year 2005 represented the moment when Interstitial Cajal-like Cells (ICLCs)—a new type of stromal cells (at that time)—were documented within the interstitium of the human pancreas [5]. This was followed by a scientifically enthusiastic time of about five years of progressive accumulation of data concerning their stromal presence in different (non-)cavitary organs [6,7,8,9,10,11,12,13,14,15,16,17,18,19]. Many immunohistochemical data indicated their phenotype (with slight expressional variations, mainly regarding their localization) and their interactions with different neighboring stromal elements (or structural elements of different origins) [7]. From the very beginning, there were many attempts to intensively document their roles and their functional meaning within the interstitium [10,13,14,15,17,19,20,21]. However, the more ICLCs research was performed, the more the idea crystalized that those cells were, in fact, a brand new and distinct type of stromal cell, rather than just a cell resembling the Interstitial Cells of Cajal. Thus, 2010 represents the year when the term telocytes (TCs), from ‘*telos*’ (gr., an individual’s most significant potential) was assigned for these cells [12]. This name conceptualizes and summarizes their most peculiar microscopic feature: the characteristic presence of telopodes (Tps), TCs’ cellular prolongations (very long, tortuous, slender, and beads-on-a-string-like). Afterward, many redundant articles repeated frequently, randomly, the well-acknowledged ultrastructural features of these Tps, i.e., their alternating successive dilated segments (podoms), their thin segments (podomers), and their spatial position in relation with interstitial structural elements, some of different origin and function. In fact, at the beginning, all these morphological findings suggested many speculations and hypotheses regarding their roles and possible primary (or secondary) functions in the homeostasis or the pathology progression of different organs (TCs being present in their interstitia).

However, analyzing the evolution of this concept of Telocytes—as new interstitial cells—it seems that it is closely following the Gartner hype cycle (as it was presented by Jackie Fenn and Mark Raskino in their book *Mastering the Hype Cycle*, in 2008). New data are still needed to move forward in defining their roles and identifying biomarkers and, moreover, to define those attributes that could promote them as players in cellular therapies for diverse pathologies. Only a few recent published data suggest that, unlike other interstitial cells, TCs seem to have lost their resistance to tamoxifen, a selective estrogen receptor modulator [22].

## 3. Skin TCs

TCs are present within the dermis of the skin [23,24]. Dermal TCs microscopically repeat almost all their well-acknowledged morphological features but also have specific distribution patterns in the reticular dermis. Herein, TCs’ distribution suggested some specific implications in skin homeostasis. The skin stem cells niche is well acknowledged. It is located within the space delimited by the hair follicle, the secretory duct of the sebaceous gland, and the hair insertion of the arrector pili muscle. TCs are part of this niche [23]. In this respect, supplemental data was provided by immunohistochemical, mRNA, and proteomics profiling of TCs [25]. However, some previously documented cases of TCs’ maldistribution and/or ultrastructural changes in some skin pathologies indicate, somewhat ambiguously (based only on IHC studies), some specific changes in TCs. Those changes were mentioned as having a direct relation with dominant pathologic conditions with respect to the underlying physiopathology of the given skin disorder [26]. However, after bleomycin treatment, the underlying dermis contains a reduced number of TCs, with swollen cell bodies with cytoplasmic vacuolization and interrupted and shortened Tps [27].

### 3.1. Location and Morphology of Skin TCs

**Figure 1:**
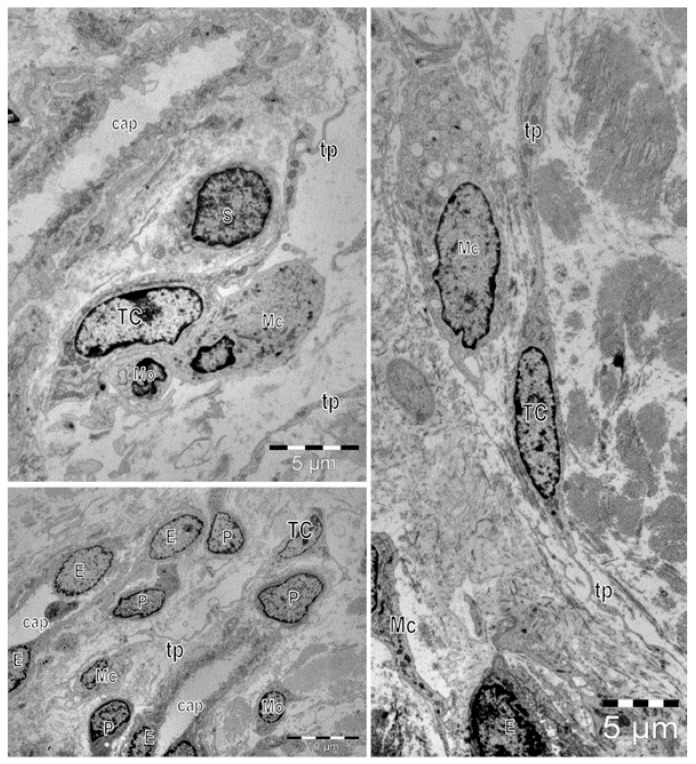
Electron microscopy images show telocytes (TCs) with characteristic long cellular prolongations with uneven caliber, named telopodes (Tps), in a human skin biopsy. TCs are mainly located around capillaries (cap). E—endothelial cell, P—pericyte. Frequently, close vicinity between TCs and other cells located in the interstitium can be observed: Schwan cells (S), macrophages (Mc), and mononuclear cells (Mo).

Within normal skin dermis (human or mammalian), TCs were frequently described in the deeper reticular dermis (in the proximity of skin adnexa), or being in close relations with connective tissue elements (blood vessels, nerve endings). For accurately attesting TCs and identifying their ultrastructural features, several electron microscopy techniques were employed [28]. Skin TCs demonstrated the typical profile defined by a small ovoidal cellular body. The cell body volume is mainly occupied by a large-for-the-body euchromatic nucleus surrounded by a delicate rim of cytoplasm that condensates scarce organelles (mitochondria, Golgi, endoplasmic reticulum, and cytoskeletal components) [12] Figure 1. The cell membrane could additionally present caveolae, the entire cell being enwrapped within an external lamina. The commonness of these morphologic features is arguable since the most particular feature of TCs is the presence of Tps, each time issued abruptly from the cell body and typically being of uncommonly great length. Tps are alternating segments of a gracile diameter (comparable to that of collagen fibrils) with widened segments that concentrate organelles (mitochondria, endoplasmic reticulum, and caveolae). In many cases, only one section plane usually accommodates a limited number of ultrastructural details and few Tps. It does not offer premises for discussing TCs’ spatial conformation. However, in semithin sections, the visible number of Tps is subsequently changing the shape of the cellular body. More advanced electron microscopy techniques (transmission electron tomography, FIB-SEM) were employed for elucidating and explaining the spatial organization of skin TCs. Apparently, due to the thinness of the sections, previously published electron micrographs for TCs showed a pattern of a cell with very long and thin cellular prolongations. The digital reconstructions of serial-focused ion beam section samples suggest, in fact, veil-like structured Tps (instead of being thread-like), mainly flattened and corresponding for the so-called polymers (thin segments) but having protuberances in their continuum (the spatial equivalents of podoms). The ultrastructure of TCs is different from that of every other cell, including myofibroblasts [29].

### 3.2. Immunohistochemical Profile of Skin TCs

Immunohistochemistry (IHC) indicates two phenotypical subsets of reticular dermis TCs, both positive for vimentin, but one positive for CD117 and the other positive for CD34 [30]. However, further IHC studies indicated positive expression of Platelet-Derived Growth Factor Receptor alpha (PDGFRα) for papillary dermis TCs. Presently, the combination of CD34 and PDGFRα markers best describes the presence of TCs in skin [28,30]. We may conclude that this association of IHC markers is individualizing TCs from other interstitial cells, such as fibroblasts (Fbs), myofibroblasts, mast cells, pericytes, macrophages, dermal dendritic cells (plasmacytoid dendritic cells, inflammatory dendritic cells), stem cells, Langerhans cells, endothelial cells, nerve cells, and melanocytes. These differences were emphasized by the analyses of the cytokine expression of TCs in comparison with other interstitial cells [31]. Despite all accepted and published morphological evidence, the immunohistochemical profile, and physiological data, there are still paralogisms in identifying and perceiving TCs and understanding their existence [22]. However, it seems that, in the proximity of epithelia, TCs show positivity for PDGFRα since it was observed in TCs from intestinal mucosa [32].

Previous comparative studies showed that skin TCs are a distinct cell population, different from fibroblasts (Fbs), the most common connective tissue cell, since they are not expressing CD90 or procollagen. These data are of keen importance to precisely identify in situ TCs or isolate TCs only for further investigations. Skin TCs localized in the proximity of hair follicles and sebaceous glands show positive expression of PGP 9.5, S100, cytokeratin-19, keratin-5, and MHC II [33]. Their distribution suggests metabolic and structural role(s), which have implications in renewal repairing processes [34,35]. Compared with skin Fbs, dermal TCs are upregulating Epithelial-Neutrophil Activating Peptide (ENA-78) and Granulocyte Chemotactic Protein 2 (GCP-2), among other differences in the 37 cytokine expression profiles, among which angiogenin, thrombopoietin, Interleukin 5 (IL 5), monocyte-chemotactic protein 3 (MCP-3), monocyte-chemotactic protein 4 (MCP-4) and Macrophage Inflammatory Proteins 3 (MIP-3) [36].

However, it was previously demonstrated in the stroma of other organs that TCs could present a diverse immunophenotypic expression [37]. Within the lamina propria of various mucosae (also connective tissue associated with epithelia), digestive or urinary, similar to the testis interstitium, TCs proved to be CD34+/ α-smooth muscle actin (αSMA), which differentiate them from the local myoepithelial with lack of expression of CD34 [38]. However, in the urinary system, TCs were found positive for α-SMA [39].

In the lamina propria of the intestinal mucosa, TCs have a positive expression of winged-helix transcription factor forkhead box l1 (FOXL1) and leucine-rich repeat-containing G-protein coupled receptor 5 (LGR5) [40]. *FOXL1* gene encodes transcription factors involved in cellular metabolism, cell proliferation, and ontogenesis [32]. On the other hand, the *LGR5* gene encodes a protein responsible for the formation of intestinal stem cells and the maintenance of their stemness during postembryonic development [41].

The c-kit receptor is essential in melanogenesis, among other melanocyte physiological processes, and its involvement in these processes is still under investigation [42]. Some recent studies showed that skin TCs also express c-kit, which could support the hypothesis that they may influence melanin production [40]. Only one study conducted on human samples from the scalp demonstrated c-kit expression in cells morphologically resembling TCs. The spatial interaction between TCs and the melanocytes at the level of the hair follicle could offer new insights into the melanogenesis, the melanization process, and new approaches to the dyschromic diseases of the skin and hairs [30]. Otherwise, in most studies conducted on human samples, skin TCs were found negative for c-kit [43].

To date, there are no reports on a specific TCs’ marker to facilitate the TCs’ identification and isolation and to bring the subject to a new level of functional studies. In vitro studies based on primary cultures of interstitial cells are usually a mixture of cell types, TCs included [44]. A selection based on a specific marker could facilitate in vitro approaches which would represent a significant step toward developing cellular therapies with TCs.

There is sufficient evidence that local transplantation of interstitial cells can promote wound healing by regulating the inflammatory stages. Some studies proved TCs to be responsible for upregulating inflammatory factors in HaCaT cells and Human Dermal Microvascular Endothelial Cells, therefore being implicated in the modulation of inflammatory events and promoting skin wound healing in experimental models [45].

So far, different techniques for isolating TCs have been proposed, including immunomagnetic microbead-based cell separation. This technique was claimed as a safe and easy way to produce pure cellular suspensions of skin TCs. By this technique, the separated cells respect the already mentioned morphological criteria for cultured TCs and their immunophenotype (positive for CD34, PDGFRα, and vimentin, and negative for CD31) [45]. This opens new perspectives on the roles of these peculiar cells and their involvement in homeostasis. However, this could prove their presumptive roles in pathology [46].

### 3.3. Skin TCs: Stromal Inter-/Intra- Relations

Within the connective tissue of the dermis, TCs are interconnected within a stromal cellular labyrinthine interstitial network either by homocellular junctions such as *puncta adherentia minima*, *recessus adherents*, or gap junctions, or heterocellular junctions connecting TCs with other stromal cells such as immune cells, nerve cells, and endothelial cells in a three-dimensional network [40,47]. Besides these physical interactions of TCs, there is also chemical/molecular communication by paracrine secretion of microRNA and signaling proteins [28,48]. These data suggest TCs’ involvement in homeostatic processes in the dermal environment. Moreover, proteins involved in the mediation of cell–cell adhesion like N-cadherin, cadherin-11, and catenin α and β were identified at the level of these junctions [30]. Communicating gap junctions (for small molecule transfer) were also reported [49,50]. These could represent the integrative role of TCs, which could receive inputs either from other interstitial cells they are contacting by point or planar contacts, by stromal synapses, or from other TCs [23,47]. In avian skin, TCs are involved in homo-cellular junctions by gap junctions, but also hetero-cellular junctions represented mainly by point contacts with melanocytes and basal keratinocytes [40]. The close interaction with interstitial immune cells (e.g., mast cells, monocytes, etc.) suggests the immunomodulatory role(s) of TCs [51]. Thus, TCs could represent immunologic players, being involved in immune scenarios of various diseases [52]. TCs can influence the activity of surrounding cells such as mast cells, lymphocytes, plasma cells, and macrophages [52], and control the local microenvironment by juxtacrine communication through molecular signals, exosomes, and multivesicular bodies. In normal skin (as in other organs) and few studied dermatologic diseases [53], evoking similar findings from myocardial infarction, TCs represent (key-)players in orchestrating the immune events that are dominating at least the early stages of lesion progression [45,54].

By paracrine secretion mediated by shedding vesicles with protein content, TCs seem to modulate their microenvironment and the activity of adjacent cells [55]. For example, the microscopic investigation of human skin samples proved their frequent association with local mast cells. This could prove their involvement in several allergic states or even skin mastocytosis [23].

### 3.4. Presumptive Roles of Skin TCs

In the normal dermis, but also skin lesions, TCs were found in the perivascular space suggesting roles in (neo)angiogenesis [28] or in blood vessel homeostasis [56]. However, by their distribution and density under several circumstances (physiological and pathological), TCs could modulate blood vessel dynamics in normal skin and different diseases [57]. Previous studies identified TCs as cellular players in angiogenesis, forming networks with the perivascular distribution. For example, in embryos, perivascular TCs have positive expression of CD34, Vascular Endothelial Growth Factor (VEGF), CD68, and matrix metalloproteinase-9 (MMP-9), suggesting their involvement in regulating angiogenesis by degrading the basement membrane [48,54]. However, considering their phenotype and secretory profile, it is also attractive to hypothesize their roles as cell players in renewing or repairing processes after skin injuries [58].

Skin TCs were demonstrated to have an endocytic function, intake pigment of hemosiderin and melanin [59], or transfer pigment to other cells [40].

**Figure 2:**
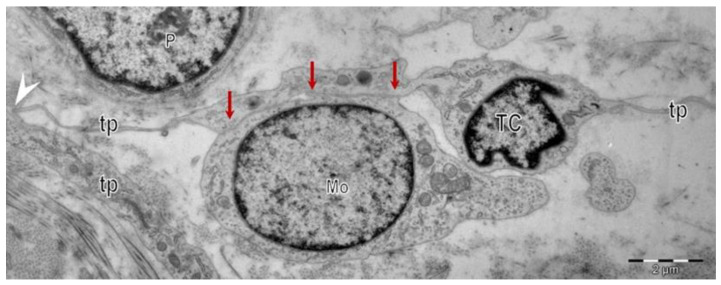
Electron microscopy images show telocytes (TCs) with telopodes (Tps) in a human skin biopsy. Heterocellular contacts (red arrows) are evident between telocytes (TCs) and the other cells in the interstitium. Homocellular junctions (arrowhead) connect the telopodes (Tps) of different telocytes (TCs) in a vast interstitial network.

However, considering the cellular interactions of TCs (as members of interstitial cellular networks), or their influencing of the local environment through their paracrine secretion or by intercellular junctions of various types (Figure 2), all previously published data suggest presumptive roles of TCs in intercellular signaling, in interstitial homeostasis, and wound healing [52,60]. The assumptions regarding these roles were mainly based on TCs’ morphology (their contacts and communications through Tps) and distribution, their secretion of extracellular vesicles and their structural ability to create 3D networks [61] (Figure 1). The roles of mechano–transduction and hormone receptors for TCs were hypothesized in the interstitium of other organs [62,63]. The ultrastructural features, cytokine/immune profile, and distribution (in networks) within the reticular dermis (in close vicinity with skin adnexa, blood vessels, and nerve endings) suggest roles as the nursing cells for epithelial stem cells, and roles in immunologic dermal pathologies by interactions with immune cells (e.g., mast cells).

Former suppositions regarding the relation between TCs and tumor-associated Fb should be assessed through an understanding of molecular and phenotypical interaction between TCs and stem cells within stem cell niches [47].

Outside the skin, in cardiac stem cell niches of the normal heart [64], in lung interstitium [65], or in the intestine [47], TCs showed contact with stem cells. These contacts were a point or planar contacts. Moreover, the arrangement pattern of TCs within the intestinal lamina propria is disrupted in inflammatory bowel disease [47]. Moreover, considering their hetero-cellular contacts (either by junctions or by soluble factors) through intercellular communication, TCs could promote wound healing at higher rates. These findings indicate a presumably close TCs cellular collaboration in skin. Thus, TCs could actively be involved in maintaining the normal skin cellular environment [23].

Moreover, TCs have positive expression of miR-21, which induces HIF-1α expression [66], hypoxia being a prerequisite for MMP-9 activation [67]. Disarrangement and reduction of TCs distribution were also documented in stem cell niches of the gastrointestinal mucosa in inflammatory pathologies. Deviant distribution of TCs may affect tissue homeostasis and regeneration capability or reverberate tissue disarrangement and malfunctioning [47].

Near another epithelium, within the lamina propria, the stem cell niches are localized near the tip of the intestinal villi [68]. TCs are active elements of these intestinal stem cell niches [32]. TCs’ malfunction or disappearance affect the extracellular matrix composition or local gene expression [69]. TCs expressing FOXL1 were found in the proximity of enterocytes. Immunohistochemistry and immune electron microscopy showed that intestinal TCs are PDGFRα and FOXL1 positive, being localized close to the basement membrane [70]. Subepithelial TCs represent the source of molecules belonging to the Wnt (Wnt ligands and Wnt inhibitors) signaling pathway [70]. Wnt signaling molecules modulate the proper differentiation of intestinal stem cells, thus involving TCs in epithelial turnover during preparatory processes [38]. The base of the crypts (also the location of stem cells) has the highest expression levels of Wnt molecules.

Moreover, it is well known that overexpression of stem cells’ Wnt signaling molecules could induce tumorigenesis [71]. In the digestive interstitium, the Fox1l positive TCs co-localize with Ng2 expression. It is well known that the *Ng2* gene defects induce failure in the differentiation of stem cells [32].

On the other hand, in the heart, TCs regulation of the WNT signaling pathway (and concomitantly other pathways) conducts cardiomyocyte differentiation and maturation, helping the integration of young cardiac muscle cells into adult heart architecture [72]. However, the impairment of maturation of Wnt proteins is followed by the decrease of stem cell activity, subsequently undermining digestive epithelial renewal [70].

## 4. Skin TCs in Dermatologic Pathology

The presence of TCs, quantitatively and qualitatively, is influenced by different skin conditions, either regular or pathophysiological. Several diseases acknowledged cellularity changes, and these studies showed various (ultra)structural changes of TCs and their spatiality with different components of the interstitium.

In systemic sclerosis, the progressive accumulation of collagen type II and III packages within the dermis (either papillary or reticular), the mucoid edema, and panniculitis, leads to disruptions of typical TCs arrangements and the architecture of normal tissue [44]. TCs are becoming less dense (in the initial stages, more prominent in the papillary dermis, and after that, in the reticular dermis). Their changes are parallel with altering the extracellular matrix and further disrupting the TCs interstitial network. The degradation of TCs integrity and their interstitial localization is progressive as the fibrotic process advances [27]. Ultrastructurally, TCs are hypoxically changed, with vacuoles within their cytoplasm containing swollen mitochondria and lipofuscin bodies. However, there is still an ongoing debate, since they could be perceived as the cause or the effect of these changes. These new TCs’ cellular pathological perspectives and their integration into pathophysiological microenvironment can dramatically change the structural considerations about these cells. However, in dermal sclerosis TCs, global intradermic distribution is reduced, and they are prominently present around skin adnexa, blood vessels, or nerve endings [23]. It will, however, be interesting to establish whether the ultrastructural changes in TCs result from the ischemia and whether TCs are affected more in comparison with other interstitial cells. Adversely, another theory stipulates that those initial ultrastructural changes of TCs could trigger the dermal deposition of over-produced collagen fibers [43].

On the other hand, psoriasis—keratinization secondary to dermal inflammation, with immunologic determinants and genetic background, with consequences over the epidermal turnover—is also featured by changes in TCs ultrastructure. The dystrophic changes include disruption of the cellular integrity (involving the cellular bodies, fragmentation of the cytoplasm and cell membrane, nuclear exclusion) and the loss of integrity of Tps with the fragmentation of their continuum at different levels. Interestingly, in microscopical studies, frequently dendritic cells were found in contact with the extruded nuclei of disintegrated TCs. This aspect suggests a series of immune reactions triggered by TCs destruction. These TCs ultrastructural changes are occurring contemporaneously with the increasing density of the dermal dendritic cells and the migration of Langerhans cells from the epidermis to the dermis [31]. Moreover, psoriasis is featured by several vascular changes, TCs being less dense in their proximity, together with the loss of contractile phenotype of smooth muscle cells [31].

Published data indicated that the increased density of TCs (positive to PDGFRα) helps improve the evolution of chronic skin wounds, reconfirming previous results regarding myocardial regeneration/repairing processes [45].

The skin carcinomas, either basal cell carcinoma or squamous cell carcinoma, consist of affected TCs less involved in heterocellular junctions, in comparison with TCs in normal tissue [73]. At the level of these junctions, TCs present inner plaques of dense electron microscopic material. Moreover, their plasma membrane could be fused (a plasma membrane particularity frequently found in tumor cells).

## 5. Skin TCs in Aging, Injury and Repair: Are TCs Key Players?

Normal skin is aging, and as a natural phenomenon, it becomes wrinkled, especially in the flexural areas, predominantly in sun-exposed areas [74]. Of course, genetics plays a role in susceptibility to all these changes. However, there are also other factors contributing to this, such as the loss of elasticity (by aging), the lowering of the fat in the fascia subcutaneous, photo-aggression (by repeated exposure to UVA), the detrimental effects of smoking, and repeated and excessive movements and contractions of the deep muscles [75,76]. Moreover, gene expression changes by age, targeting adipose tissue, blood, and skin [77].

It is already widely accepted that there are excellent outcomes (in terms of time and morphological remodelling), at least for the skin, of using platelet rich plasma (PRP) for different therapeutic reasons. Usually, the recovery time is significantly shortened, and the positive results are unequivocally visible. PRP benefits are almost dogmatically acknowledged to be exclusively produced due to the activation of only fibroblasts by those granules released by platelets (secondary, after platelets activation). Furthermore, it is well acknowledged that PRP treatments represent an act of regenerative medicine, in fact, PRP represent an autologous administration of plasma rich in plasmatic proteins, previously centrifuged to remove the red blood cells, but preserving the platelets and their inner granules. Casual studies previously indicated that skin-administered PRP activates Fb (cells with solely structural roles), thus increasing the synthesis of collagenic and non-collagenic proteins. However, sensitive, specific data regarding the exact sequence of cellular activation, the molecular fundaments that underline the PRP-induced structural benefits, and the succession of activated cells, are still limited and equivocal. Thus, it will become interesting to also consider TC’s presence and cellular response after administering different skin boosters or local skin procedures that were already documented for their clinical benefits.

In case of common skin injuries (erosion, cut, burn, etc.), both the epidermis and dermis could be intercepted. The skin repairing/regeneration process is an excellent orchestration of dermal/epidermal cell types. Cellular heterogeneity represents a critical element of skin healing progression [78,79]. Irrespective of their etiology, chronic and acute skin wounds imply inflammation. Naturally, the resolving of skin wounds requires vascular support during all four stages, which develops synchronously with dermal and epidermal repairing through synthetic fibroblast activation and keratinocyte proliferation and differentiation [45]. Healing stages are different for each skin layer (epithelium and connective tissues) and, at every specific moment, are featured by different specific aspects of the repairing and regeneration [80,81,82,83]. The repairing process for the epidermis is more straightforward and mainly consists of the migration of keratinocytes (within the first few hours of the healing process) mediated by fibronectin and followed by the proliferation of epidermal cells. 

However, for a deep wound affecting the integrity of the dermis, the pivotal events triggering the reparatory processes are represented by the haemorrhage and clot formation [84]. The fibrinogen is polymerized into fibrin and consequently stabilized into fibronectin binding. Thus, the gel consisting of fibrin and fibronectin plugs the tissue defect and acts like a sponge for platelets to accumulate [85]. Some well-defined stages of wound development follow the skin injury and the haemostasis phase. The inflammatory cellular infiltration is triggered in the first hours by the accumulation of neutrophils. This is followed by the migration of macrophages that initiate their phagic roles, further attracting the neighboring fibroblasts, and stimulating all to produce a matrix. The transition to the proliferative phase and granulation tissue formation represents a crucial step in wound development [86]. The granulation phase is featured by (neo)angiogenesis within the fibrin gel, matrix formation, and restoration of the vascular network [87]. This collagen production phase is defined by the tensile strength within the wound and by the amount and orientation of collagen fibers [88]. However, imperfect collagen production during this phase could increase collagen production and accumulate large collagen deposits, resulting in a bulky clinical scar, the keloid [89].

All these cellular events are orchestrated by various chemical signals of different origins. The growth factors (either competence or progression growth factors) are mainly provided by platelets (and some by fibroblasts) and can induce the cell cycle in stem cells, but they are also inducing the mitogenic activity of the cells at the wound level [90]. The typical competence growth factors are platelet-derived growth factor (PDGF) and fibroblast growth factor (FGF). The epidermal growth factor (EGF) and somatomedins are progression growth factors. On the other hand, the cytokines produced by the inflammatory cells (interleukins, monokines, lymphokines, and interferons) have mainly regulatory functions. Cytokines are proteins of low molecular weight than could act in an autocrine and paracrine fashion [91].

PDGF is a dimeric glycoprotein that could have different sources. The interaction between PDGF and its receptor is followed by the activation of *c-fos* and *c-myc* (two protooncogenes). PDGF is mainly synthesized and stored within the alpha granules of the thrombocytes, and it can be released by activating the platelets [92]. Otherwise, PDGF could be produced by other cells like endothelial cells, macrophages, and smooth muscle cells. Besides the mitogenic role, it could be chemotactic for mesenchymal cells and vasoconstrictors, increase the number of LDL receptors, increase the secretion of prostaglandins, induce changes in cellular shape, and play a crucial role(s) in (neo)angiogenesis [93].

Previous studies demonstrated that in experimental acute myocardial infarction, the border zone of myocardial infarction lesion (metabolically and immunologically, the most active area), at given successive time points, is dominated by different types of cells: inflammatory cells, myofibroblasts, fibroblasts, and TCs. However, 30 days after myocardial infarction, the local cellular scenery is mastered by TCs, mainly in close spatial interactions with blood vessels (including the new-formed blood vessels).

Thus, further studies must be conducted to elucidate the involvement of skin TCs in reparatory/regeneration processes secondary to an acute injury of the skin, or a chronic skin defect, especially after administering growth factors at the lesion site (like PRP). It is possible that such growth factors augment tissue regeneration by enhancing the presence, distribution, density, and activity of skin TCs. Moreover, such results could be further extrapolated and investigated.

## 6. Conclusions

TCs represent an entirely distinct cellular population of the dermis, they are being distinct from other stromal cells. To date, skin TCs have been shown to possess a specific ultrastructural profile, different from any other known cell, and an individual immunohistochemical profile, with characteristically double positivity for CD34 and PDGFRα. Their distribution and density are higher within the reticular dermis than papillary dermis, and they are forming in a stromal network by intercellular junctions (either in between TCs only or in between TCs and other stromal cells). Due to all these structural and phenotypical characteristics, it is tempting to ascribe them peculiar roles in skin homeostasis, or in the pathophysiological progression of skin disorders. An attractive research path is to consider TCs (among other cells) as cellular players for skin grafts, transplanted or artificially reconstructed skin substitutes [44]. Therefore, identification of only one biomarker (or a panel of biomarkers) that can leverage skin TCs’ involvement in normal/pathologic conditions should become a further distinct research direction. 

A large body of data indicated that at least few documented skin pathologies are featured, *inter alia*, by the disturbance of the regular tissue distribution of dermal TCs. Furthermore, the clinical and/or structural recovery of these dermatological conditions feature (or, even, are determined by) the rehabilitation of dermal TCs’ presence in their previous distribution. Thus, one question that can be raised as to whether TCs are one of the players in the repairing process, or if their recovery is only a consequence of the tissue repair complex cellular orchestration. However, considering the presence of TCs in skin stem cell niches, in respect to the previously published data showing TCs’ involvement in myocardial regeneration (and their potential in nursing cardiac progenitor cells) and liver regeneration, it is tempting to hypothesize they may serve the same roles in the skin regeneration/repair processes. Recent studies have already proved that the supplementation of local TCs by transplant can reduce local inflammation [45]. However, further studies should be conducted in deciphering TCs’ implication (if any) in the morphological substrate of those repairing and/or rejuvenation processes that feature wound healing or the cosmetic improvement of senescent skin.

One line of research could derive from the new minimally invasive procedure, platelet rich plasma (PRP) injection. This is improving the tissue regeneration/repair in different dermatological conditions (from skin scars to hair loss) and is usually followed by a smooth local recovery. However, there are no studies on dynamic cellular and molecular changes regarding TCs after such treatment.

The skin is one of the most accessible organs that could offer a proof-of-concept for the TCs’ involvement in diseased or injured tissue and thus could offer research insights into other diseases and potential new therapeutic approaches with multiple medical, social, economic, and social benefits.
